# Association Between Sitting Time and Cardiometabolic Risk Factors After Adjustment for Cardiorespiratory Fitness, Cooper Center Longitudinal Study, 2010–2013

**DOI:** 10.5888/pcd13.160263

**Published:** 2016-12-29

**Authors:** Carolyn E. Barlow, Kerem Shuval, Bijal A. Balasubramanian, Darla E. Kendzor, Nina B. Radford, Laura F. DeFina, Kelley Pettee Gabriel

**Affiliations:** 1 The Cooper Institute, Dallas, Texas; 2 University of Texas Health Science Center at Houston School of Public Health — Dallas Campus, Dallas, Texas; 3 Department of Intramural Research, American Cancer Society, Atlanta, Georgia; 4 University of Texas Southwestern Medical Center — Harold C. Simmons Cancer Center, Dallas, Texas; 5 Department of Family and Preventive Medicine, University of Oklahoma Health Sciences Center, Oklahoma City, Oklahoma; 6 Oklahoma Tobacco Research Center, Stephenson Cancer Center, Oklahoma City, Oklahoma; 7 Cooper Clinic, Dallas, Texas; 8 University of Texas Health Science Center at Houston School of Public Health — Austin Campus, Austin, Texas.

## Abstract

**Introduction:**

Objective estimates, based on waist-worn accelerometers, indicate that adults spend over half their day (55%) in sedentary behaviors. Our study examined the association between sitting time and cardiometabolic risk factors after adjustment for cardiorespiratory fitness (CRF).

**Methods:**

A cross-sectional analysis was conducted with 4,486 men and 1,845 women who reported daily estimated sitting time, had measures for adiposity, blood lipids, glucose, and blood pressure, and underwent maximal stress testing. We used a modeling strategy using logistic regression analysis to assess CRF as a potential effect modifier and to control for potential confounding effects of CRF.

**Results:**

Men who sat almost all of the time (about 100%) were more likely to be obese whether defined by waist girth (OR, 2.61; 95% CI, 1.25–5.47) or percentage of body fat (OR, 3.33; 95% CI, 1.35–8.20) than were men who sat almost none of the time (about 0%). Sitting time was not significantly associated with other cardiometabolic risk factors after adjustment for CRF level. For women, no significant associations between sitting time and cardiometabolic risk factors were observed after adjustment for CRF and other covariates.

**Conclusion:**

As health professionals struggle to find ways to combat obesity and its health effects, reducing sitting time can be an initial step in a total physical activity plan that includes strategies to reduce sedentary time through increases in physical activity among men. In addition, further research is needed to elucidate the relationships between sitting time and CRF for women as well as the underlying mechanisms involved in these relationships.

**MEDSCAPE CME**

Medscape, LLC is pleased to provide online continuing medical education (CME) for this journal article, allowing clinicians the opportunity to earn CME credit.

This activity has been planned and implemented through the joint providership of Medscape, LLC and *Preventing Chronic Disease*. Medscape, LLC is accredited by the American Nurses Credentialing Center (ANCC), the Accreditation Council for Pharmacy Education (ACPE), and the Accreditation Council for Continuing Medical Education (ACCME), to provide continuing education for the healthcare team.

Medscape, LLC designates this Journal-based CME activity for a maximum of 1.00 ***AMA PRA Category 1 Credit(s)™***. Physicians should claim only the credit commensurate with the extent of their participation in the activity.

All other clinicians completing this activity will be issued a certificate of participation. To participate in this journal CME activity: (1) review the learning objectives and author disclosures; (2) study the education content; (3) take the post-test with a 75% minimum passing score and complete the evaluation at http://www.medscape.org/journal/pcd; (4) view/print certificate.

**Release date: December 29, 2016; Expiration date: December 29, 2017**

**Learning Objectives**

Upon completion of this activity, participants will be able to:

1. Distinguish the prevalence of sedentary behavior among US adults
2. Evaluate the effect of sitting time on cardiometabolic risk factors among adults
3. Identify the cardiometabolic measure most associated with prolonged sitting time

**EDITOR**

Caran Wilbanks

Editor, Preventing Chronic Disease

Disclosure: Caran Wilbanks has disclosed the following relevant financial relationships:

Other: Partner is employed by McKesson Corporation

**CME AUTHOR**

Charles P. Vega, MD

Health Sciences Clinical Professor of Family Medicine, University of California, Irvine, California

Disclosure: Charles P. Vega, MD, has disclosed the following relevant financial relationships:

Served as an advisor or consultant for: Allergan, Inc.; McNeil Consumer Healthcare

Served as a speaker or a member of a speakers bureau for: Shire

**AUTHORS**

Carolyn E. Barlow, PhD

The Cooper Institute; University of Texas Health Science Center at Houston School of Public Health -- Dallas Campus, Dallas, Texas

Disclosure: Carolyn E. Barlow, PhD, has disclosed no relevant financial relationships.

Kerem Shuval, PhD

Department of Intramural Research, American Cancer Society, Atlanta, Georgia

Disclosure: Kerem Shuval, PhD, has disclosed no relevant financial relationships.

Bijal A. Balasubramanian, MBBS, PhD

University of Texas Health Science Center at Houston School of Public Health -- Dallas Campus; UT Southwestern -- Harold C. Simmons Cancer Center, Dallas, Texas

Disclosure: Bijal A. Balasubramanian, MBBS, PhD, has disclosed no relevant financial relationships.

Darla E. Kendzor, PhD

Department of Family and Preventive Medicine, University of Oklahoma Health Sciences Center; Oklahoma Tobacco Research Center, Stephenson Cancer Center, Oklahoma City, Oklahoma

Disclosure: Darla E. Kendzor, PhD, has disclosed no relevant financial relationships.

Nina B. Radford, MD

Cooper Clinic, Dallas, Texas

Disclosure: Nina B. Radford, MD, has disclosed no relevant financial relationships.

Laura F. DeFina, MD

The Cooper Institute, Dallas, Texas

Disclosure: Laura F. DeFina, MD, has disclosed no relevant financial relationships.

Kelley Pettee Gabriel, PhD

University of Texas Health Science Center at Houston School of Public Health -- Austin Campus, Austin, Texas

Disclosure: Kelley Pettee Gabriel, PhD, has disclosed no relevant financial relationships.

## Introduction

Prolonged sitting time characterizes the daily lifestyle patterns of most people living in developed countries (1). Estimates of median reported sitting time for US adults range between 6.5 to 8 hours per day (2). Objective estimates, based on waist-worn accelerometers, indicate that adults spend over half their day (55%) in sedentary behaviors (3). Several studies demonstrate direct, independent associations between sedentary behavior and cardiometabolic risk factors such as adiposity and fasting blood glucose level after adjustment for the beneficial effect of moderate-intensity to vigorous-intensity physical activity (MVPA), accumulated mostly during leisure or discretionary periods of the day (4). However, within a 24-hour period, people spend a significant proportion of waking hours in sedentary behaviors or light-intensity physical activities relative to time spent in MVPA (1). Therefore, investigators recently argued that accounting for an individual’s total physical activity level during the entire waking period, not just during isolated segments of the day (eg, time spent sitting or time spent highly active), is essential to understanding the complex relationships between physical activity behavior and cardiometabolic risk factors (5). Furthermore, objectively measured total activity level per day appears to be more strongly associated with cardiometabolic risk factors than is MVPA per day (6).

Given that cardiorespiratory fitness (CRF) reflects a person’s habitual physical profile and overall general health, the primary goal of our study was to determine whether among adult men and women time spent sitting was associated with elevated levels of waist girth, body mass index, body fat percentage, total cholesterol, low-density lipoprotein (LDL) cholesterol, triglycerides, glucose, and resting systolic blood pressure; low levels of high-density lipoprotein (HDL) cholesterol; and the presence of metabolic syndrome. Secondary goals were to 1) examine whether CRF confounded or modified the associations between sitting time and cardiometabolic risk factors and 2) explore whether the role of CRF differed by sex.

## Methods

Participants included in this cross-sectional analysis received a preventive medical examination at the Cooper Clinic in Dallas, Texas, during 2010 through 2013 and provided written consent to participate in the Cooper Center Longitudinal Study (CCLS). Participants in CCLS are generally healthy and self-referred or referred by their employers to the Cooper Clinic for preventive medical examinations that include a physician-administered medical examination, fasting laboratory studies, body composition measurements, and a maximal treadmill graded exercise test. For our analysis, to eliminate the potential for a disease condition that could affect the exposure of interest (eg, a stroke may result in increased sitting time), participants were excluded if they reported a personal history of cardiovascular disease (n = 51), stroke (n = 27), or diabetes (n = 582) or if they did not reach 85% of their predicted maximal heart rate on the treadmill test (n = 137). Participants were also excluded if their data for some covariates were missing (n = 332). These criteria resulted in an analytic sample of 1,845 women and 4,486 men aged 20 to 79 years. Each year, the Cooper Institute’s institutional review board reviewed and approved the overall study. Our study also received exempt status from the University of Texas Health Science Center at Houston’s Committee for the Protection of Human Subjects.

Sitting time was based on participants’ responses to a question on the medical history questionnaire completed before their clinical examination. The sitting question, derived from the Canada Fitness Survey (7), assessed the proportion of time spent sitting during work, school, and housework during waking hours on a typical day. Response options were 1) almost none of the time (about 0%), 2) approximately one-quarter of the time (about 25%), 3) approximately half of the time (about 50%), 4) approximately three-quarters of the time (about 75%), and 5) almost all of the time (about 100%).

### Cardiometabolic risk factors (primary dependent measures)

Body mass index (BMI, kg/m^2^) and body composition measurements (% body fat, waist girth) were measured during the preventive medical examination. These measurements were taken according to standard procedures by trained technicians and described previously (8). Briefly, BMI was computed as weight in kilograms divided by height in meters squared measured on a stadiometer and a standard physician’s scale. Participants with BMI of 30 kg/m^2^ or higher were classified as obese (9). Waist girth (cm) was measured with a plastic tape at the level of the umbilicus following a normal exhalation. An elevated waist girth for men was 102 cm or greater and for women was 88 cm or greater (10). Percentage of body fat was determined by measuring 7 skinfold sites (axilla, chest, abdomen, triceps, hip, thigh, and back) with calipers and inserting the sum of these skinfold measurements in a generalized body density equation to estimate percentage of body fat (11). Sex-specific cut points of percentage of body fat (<25% or ≥25% for men and <32% or ≥32% for women) were used to classify patients as obese (12).

Serum samples taken after patients fasted for 12 hours were analyzed for lipids by using automated bioassays in accordance with standard procedures. Elevated lipid levels were defined by using the following cut points: total cholesterol higher than 200 mg/dL; LDL cholesterol higher than 100 mg/dL; HDL cholesterol less than 40 mg/dL for men and less than 50 mg/dL for women; triglycerides 150 mg/dL or higher; and fasting blood glucose 100 mg/dL or higher (10).

Resting blood pressure was auscultated as the first and fifth Korotkoff sounds according to a standard sphygmomanometer protocol (13). Elevated blood pressure was defined as a systolic blood pressure 130 mm Hg or higher or diastolic blood pressure 85 mm Hg or higher, or both (10).

Using the criteria of the American Heart Association and the National Heart, Lung, and Blood Institute, we defined metabolic syndrome as meeting 3 or more of the following criteria: abdominal obesity (waist girth: ≥102 cm for men and ≥88 cm for women); high triglycerides (≥150 mg/dL); low HDL (<40 mg/dL for men and <50 mg/dL for women); high blood pressure (systolic blood pressure ≥130 mm Hg, or diastolic blood pressure ≥85 mm Hg, or physician-diagnosed history of hypertension); and high glucose (fasting blood glucose ≥100 mg/dL or physician-diagnosed history of high glucose) (10).

### Covariates

CRF was assessed by using the time to complete a treadmill-graded exercise test and the modified Balke protocol described previously (14). Duration on the treadmill is highly correlated with measured oxygen consumption (VO_2_) (r = 0.92 for men [15] and r = 0.94 for women [16]). A value for maximal metabolic equivalent of tasks (METs) was estimated from the final speed and grade of the treadmill test (17).

Participants were asked to report the frequency and duration of 11 specific physical activity types: walking, running, treadmill, swimming, stationary cycling, bicycling, elliptical, aerobic dance, racket sports, vigorous sports, and other activity. These 11 activity types represent high-intensity MVPA. Summary estimates were computed by weighting the product of the reported frequency and duration (in minutes per week [min/wk^−1^]) by a standardized estimate of the MET of each activity type (18), which was then summed across all activities performed. The leisure-time physical activity estimate was expressed as a log transformation of MET/min/wk^−1^.

On the basis of literature, we included additional covariates from the medical history questionnaire: age, sex, alcohol consumption, and smoking status. Alcohol consumption was calculated as the combined number of drinks per week of beer, wine, and hard liquor. Smoking status was categorized as current smoker or nonsmoker based on self-reported behavior. Three variables were created to indicate current medication use (yes/no) for hypertension, diabetes, or hyperlipidemia; a fourth variable, hormone replacement therapy, was created for women only. Medication use was reported by the patient to the study physician who conducted the medical examination.

### Statistical analysis

Descriptive characteristics of the study sample are presented by sex and for the total sample. To examine crude associations, we tested for linear trends reflecting the prevalence of each outcome for each sex across increasing categories of self-reported sitting time (ie, about 0% of the time to about 100% of the time). First, the potential effect modification of CRF on self-reported sitting time and each cardiometabolic risk factor was explored with the addition of an interaction term to a logistic regression model in which sitting time and CRF were used to predict each outcome. Next, CRF was added to the fully adjusted model to control for confounding effects after we determined that the effect size increased more than 10% with its inclusion in the fully adjusted model. Results are presented for each risk factor regressed against self-reported sitting time 1) adjusted for age (y) (model A); 2) adjusted for age and cardiorespiratory fitness (METs) (model B); and 3) adjusted for all covariates in model B and for self-reported physical activity (MET-minutes per week), alcohol consumption (drinks per week), smoking status (yes/no), waist girth (in models with lipids, glucose, or blood pressure as the outcome), and medication use associated with the outcome (model C). The presence of multicollinearity between self-reported physical activity and CRF was assessed and found to be weakly correlated (r = 0.34). Analyses were performed using SAS/STAT version 9.4 (SAS Institute, Inc). All significance testing was 2-sided with a *P* value of less than .05 considered significant.

## Results

The average age of the analytic sample (n = 6,331) was 50.7 (SD 10.0) years old and consisted of mostly men (71%) (Table 1). Eight percent of patients reported current smoking. Alcohol consumption was moderate (median [25th, 75th percentile], 4 [1, 9] drinks per week). A higher percentage of men (41%) than women (13%) reported sitting most or all of the time (≥75% of the time) during a usual day. The average CRF level was 11.6 (SD 2.2) METs for men and 9.8 (SD 1.9) METs for women.

**Table 1:** Selected Characteristics of Participants in the Cooper Center Longitudinal Study of Sitting Time and Cardiometabolic Risk Factors, by Sex, 2010–2013^a^

Characteristic	Men	Women	Total
**N**	4,486	1,845	6,331
**Age, y**	51.2 (9.8)	49.4 (10.4)	50.7 (10.0)
**Waist girth, cm**	94.2 (10.9)	77.4 (10.8)	89.3 (13.3)
**Elevated waist girth**	21	16	19
**BMI, kg/m^2^**	27.8 (4.7)	24.6 (5.0)	26.9 (5.0)
**Obese (BMI ≥30)**	23	11	20
**Percentage of body fat**	22.0 (5.5)	25.5 (6.3)	23.0 (6.0)
**Elevated body fat^b^**	18	10	16
**Total cholesterol, mg/dL^b^**	185.1 (36.4)	194.8 (34.3)	188.0 (36.0)
**Total cholesterol >200 mg/dL^b^**	32	42	35
**LDL cholesterol, mg/dL^b^**	108.4 (33.1)	105.5 (30.2)	107.6 (32.3)
**LDL cholesterol >100 mg/dL^b^**	58	53	56
**Use of lipid lowering medication^b^**	31	12	26
**HDL cholesterol, mg/dL^b^**	53.1 (14.8)	70.3 (18.9)	58.0 (17.9)
**HDL cholesterol <40 mg/dL for men and <50 mg/dL for women^b^**	12	17	15
**Triglycerides, mg/dL^b^**	118.2 (58.1)	95.3 (48.1)	111.5 (56.3)
**Triglycerides ≥150 mg/dL^b^**	22	12	19
**Glucose, mg/dL^b^**	95.6 (9.8)	90.2 (8.5)	94.0 (9.7)
**Glucose ≥100 mg/dL^b^**	27	11	23
**Resting SBP, mm Hg^b^**	119.5 (12.0)	111.6 (13.1)	117.2 (12.9)
**Resting DBP, mm Hg^b^**	80.0 (8.9)	75.2 (8.4)	78.6 (9.0)
**Blood pressure ≥130/85 mm Hg^b^**	34	17	29
**Use of hypertension medication^b^**	25	13	22
**Metabolic syndrome^b^**	15	6	12
**Time spent sitting^b^^,^ c**			
About 0%	11	17	15
About 25%	21	37	32
About 50%	27	34	32
About 75%	29	11	16
About 100%	12	2	5
**Cardiorespiratory fitness (METs) b**	11.6 (2.2)	9.8 (1.9)	11.1 (2.3)
**Physical activity (MET-minutes/week), median (25th, 75th percentile)^b^**	960 (382, 1,799)	892 (255, 1,750)	960 (340, 1,785)
**Current smoker^b^**	10	3	8
**Alcohol intake (drinks/wk), median (25th, 75th percentile)^b^**	5 (2, 10)	3 (1, 7)	4 (1,9)

Abbreviation: BMI, body mass index; DBP, diastolic blood pressure; HDL, high-density lipoprotein; LDL, low-density lipoprotein; METs, metabolic equivalent of tasks; SBP, systolic blood pressure.

a Values are mean (SD) or percentage of participants with the characteristic unless otherwise noted.

b Information was available for a subset of the dataset. Men, n = 2,816; women, n = 1,140; total, n = 3,956.

c Response options were 1) almost none of the time (about 0%), 2) approximately one-quarter of the time (about 25%), 3) approximately half of the time (about 50%), 4) approximately three-quarters of the time (about 75%), and 5) almost all of the time (about 100%).

For men, high self-reported sitting time was significantly associated with high prevalence of cardiometabolic risk factors, including elevated waist girth, percentage of body fat, and obesity (all *P* for linear trend < .05) (Table 2). No associations were observed for the other risk factors or metabolic syndrome. Similarly, for women, high self-reported sitting time was significantly associated with high prevalence of elevated waist girth and percentage of body fat, obesity, and metabolic syndrome (all *P* for linear trend < .001). In addition, the more women sat, the higher their levels of triglycerides and the lower their levels of HDL cholesterol (both *P* for linear trend < .001). For women, no associations were observed between self-reported sitting time and total cholesterol, LDL cholesterol, glucose, or blood pressure.

**Table 2:** Percentage of Participants With Detrimental Levels of Cardiometabolic Risk Factors According to Sitting Time Categories, Cooper Center Longitudinal Study, 2010–2013

Characteristic	Sitting Time^a^					
	About 100%	About 75%	About 50%	About 25%	About 0%	*P* Value for Trend
**Men**						
n	757	1,669	1,511	469	80	—
Elevated waist girth (≥102 cm)	24	21	21	19	13	.014
Obese (BMI ≥30)	28	23	22	19	15	<.001
Elevated percentage of fat (≥25%)	36	29	27	22	17	<.001
Elevated total cholesterol (>200 mg/dL)	32	33	33	31	34	.97
Elevated LDL cholesterol (>100 mg/dL)	58	59	58	62	55	.052
Low HDL cholesterol (<40 mg/dL)	18	17	16	16	10	.14
Elevated triglycerides (≥150 mg/dL)	24	22	22	21	21	.26
Elevated glucose (≥100 mg/dL)	26	28	27	31	27	.29
Elevated blood pressure (≥130/85 mm Hg)	19	16	17	17	22	.56
Metabolic syndrome	16	15	15	13	11	.22
**Women**						
n	204	390	496	535	220	—
Elevated waist girth (≥88 cm)	26	18	16	13	10	<.001
Obese (BMI ≥30)	23	14	11	7	7	<.001
Elevated percentage of fat (≥32%)	23	22	13	13	11	<.001
Elevated total cholesterol (>200 mg/dL)	43	39	43	41	44	.49
Elevated LDL cholesterol (>100 mg/dL)	56	52	54	53	52	.69
Low HDL cholesterol (<50 mg/dL)	4	4	2	2	1	<.001
Elevated triglycerides (≥150 mg/dL)	16	14	11	10	7	<.001
Elevated glucose (≥100 mg/dL)	12	11	12	11	10	.63
Elevated blood pressure (≥130/85 mm Hg)	9	7	7	6	6	.30
Metabolic syndrome	11	6	5	5	2	<.001

Abbreviations: —, not applicable; BMI, body mass index; HDL, high density lipoprotein; LDL, low density lipoprotein.

a Response options were 1) almost none of the time (about 0%), 2) approximately one-quarter of the time (about 25%), 3) approximately half of the time (about 50%), 4) approximately three-quarters of the time (about 75%), and 5) almost all of the time (about 100%).

Next, we assessed the role of CRF as an effect-modifying variable by adding a self-reported sitting time × CRF interaction term to the models for each separate cardiometabolic outcome. This interaction term was not significant for any of the cardiometabolic risk factors after adjustment for covariates for either men or women (all *P* > .05).

For men, the crude associations that were observed between self-reported sitting time and each measure of adiposity remained significant after covariate adjustment, including CRF (Table 3). More specifically, in model C, men who reported sitting about 100% of the time were more than twice as likely to be obese whether defined by waist girth (OR, 2.61; 95% CI, 1.25–5.47), or percentage of body fat (OR, 3.33; 95% CI, 1.35–8.20) relative to men who sat about 0% of the time. Similar to the results for men, associations between self-reported sitting time and each measure of adiposity were seen among women (Table 3) when adjusted for age (model A). However, unlike men, when CRF was added to the model (model C), these associations for women were no longer significant. Self-reported sitting time was not associated with the remaining risk factors among men or women (Appendix).

**Table 3:** Association Between Sitting Time and the Prevalence of Detrimental Levels of Cardiometabolic Risk Factors, Men and Women, Cooper Center Longitudinal Study, 2010–2013

Characteristic/Model^b^	Sitting Time^a^				
	About 100%, Odds Ratio (95% CI)	About 75%, Odds Ratio (95% CI)	About 50%, Odds Ratio (95% CI)	About 25%, Odds Ratio (95% CI)	About 0%
**Men**					
**Elevated waist girth (≥102 cm)**					
Model A	2.17 (1.12–4.20)	1.77 (0.92–3.39)	1.73 (0.90–3.31)	1.49 (0.76–2.93)	1 [Reference]
Model B	2.54 (1.29–5.00)	2.09 (1.08–4.07)	1.95 (1.00–3.80)	1.60 (0.80–3.20)	1 [Reference]
Model C	2.61 (1.25–5.47)	2.27 (1.10–4.69)	2.09 (1.01–4.32)	1.68 (0.79–3.58)	1 [Reference]
**Body mass index**					
Model A	2.17 (1.15–4.10)	1.72 (0.92–3.21)	1.57 (0.84–2.94)	1.31 (0.68–2.52)	1 [Reference]
Model B	2.53 (1.32–4.84)	2.01 (1.07–3.81)	1.76 (0.92–3.33)	1.38 (0.70–2.69)	1 [Reference]
Model C	2.51 (1.24–5.05)	2.08 (1.05–4.14)	1.74 (0.88–3.47)	1.34 (0.65–2.76)	1 [Reference]
**Elevated percentage of body fat (≥25%)**					
Model A	3.38 (1.47–7.76)	2.48 (1.09–5.62)	2.11 (0.92–4.80)	1.40 (0.59–3.31)	1 [Reference]
Model B	3.74 (1.61–8.67)	2.78 (1.21–6.40)	2.28 (0.99–5.24)	1.54 (0.64–3.68)	1 [Reference]
Model C	3.33 (1.35–8.20)	2.66 (1.09–6.48)	2.06 (0.84–5.02)	1.22 (0.48–3.11)	1 [Reference]
**Women**					
**Elevated waist girth (≥88 cm)**					
Model A	3.54 (2.06–6.10)	2.20 (1.32–3.67)	1.74 (1.05–2.87)	1.29 (0.78–2.16)	1 [Reference]
Model B	3.07 (1.75–5.41)	1.94 (1.14–3.29)	1.61 (0.96–2.71)	1.30 (0.77–2.20)	1 [Reference]
Model C	1.77 (0.95–3.29)	1.30 (0.73–2.30)	1.21 (0.69–2.11)	1.11 (0.63–1.95)	1 [Reference]
**Body mass index**					
Model A	4.04 (2.18–7.51)	2.27 (1.25–4.12)	1.63 (0.90–2.96)	1.11 (0.60–2.05)	1 [Reference]
Model B	3.51 (1.85–6.66)	1.95 (1.05–3.60)	1.47 (0.80–2.71)	1.07 (0.57–2.00)	1 [Reference]
Model C	1.63 (0.79–3.35)	1.06 (0.54–2.10)	0.91 (0.46–1.79)	0.76 (0.38–1.51)	1 [Reference]
**Elevated percentage of body fat (≥32%)**					
Model A	2.58 (1.29–5.15)	2.25 (1.21–4.18)	1.23 (0.65–2.31)	1.26 (0.68–2.33)	1 [Reference]
Model B	1.98 (0.97–4.06)	1.83 (0.97–3.48)	1.09 (0.57–2.08)	1.21 (0.64–2.27)	1 [Reference]
Model C	1.15 (0.51–2.60)	1.12 (0.54–2.33)	0.85 (0.41–1.76)	1.03 (0.51–2.11)	1 [Reference]

a Response options were 1) almost none of the time (about 0%), 2) approximately one-quarter of the time (about 25%), 3) approximately half of the time (about 50%), 4) approximately three-quarters of the time (about 75%), and 5) almost all of the time (about 100%).

b Model A, adjusted for age; model B, adjusted for age and cardiorespiratory fitness (metabolic equivalent of tasks [METs]); and model C, adjusted for all covariates in model B plus physical activity (MET-minutes per week), alcohol consumption (drinks per week), current smoking status, waist girth (in models with lipids, glucose, or blood pressure as the outcome), and hormone replacement therapy (women only).

## Discussion

Our findings suggest that prolonged sitting is associated with high levels of adiposity among men even after accounting for their CRF level. However, this relationship between self-reported sitting time and adiposity was not found for women. Furthermore, for men, other cardiometabolic risk factors (elevated lipids, blood glucose, triglycerides, and blood pressure; low levels of HDL; and the presence of metabolic syndrome) were not significantly associated with sitting time. For women, self-reported sitting time was not associated with any individual cardiometabolic risk factor or the presence of metabolic syndrome.

Previous cross-sectional studies report significant associations between sedentary behavior and various cardiometabolic risk factors after controlling for MVPA (19,20). However, these studies probably suffer from incomplete ascertainment of an individual’s exposure to physical activity given that only a small portion of the day was examined (ie, 3% of their day assuming 30 minutes per day of MVPA during 16 waking hours), which in turn could explain the significant associations found in published study results. In their study of National Health and Nutrition Examination Survey (NHANES) participants, Maher et al found high-sensitivity C-reactive protein and triglycerides to be the only cardiometabolic risk factors associated with sedentary behavior when controlling for total physical activity time as assessed with accelerometers, which produce information about activity throughout the day (5). Although these associations reached statistical significance, the relationships were weak and not of clinical significance. In addition, a prospective study of men in the CCLS cohort found that prolonged TV viewing and time spent in a car were detrimentally linked only to a marker of insulin sensitivity (but not to other cardiometabolic risk factors) when CRF was taken into account (21).

Similar to the results from NHANES (5), our study found that self-reported sitting time was not associated with cardiometabolic risk factors other than obesity for men when reported physical activity level or cardiorespiratory fitness level are taken into account. However, little evidence exists of studies having explored the potential role of CRF in the relationship between estimates of total sitting time and cardiometabolic risk factors. The role of CRF appeared to differ for men and for women, and this finding also deserves further study. More specifically, for men, CRF confounded the relationship between sitting time and cardiometabolic risk factors: men had higher levels of muscle mass (70 kg) than women (50 kg), which might protect men against the adverse effect of prolonged sitting on lipids, glucose, and blood pressure, but not against the accumulation of body fat. For women, CRF may have confounded the effect of sitting time on some risk factors, but it did not modify this relationship. A previous cross-sectional study of the CCLS cohort found that the more women sat, the lower their fitness level (22). Therefore, high levels of time sitting during the day could lower fitness levels and lower total daily caloric expenditures, which could lead to increases in women’s body fat. For different levels of CRF, we found no sex-related difference in the relationship between sitting time and cardiometabolic risk factors.

Our study findings have public health and clinical implications: they indicate that, among men, increased self-reported long sitting time is related to a higher likelihood of obesity. These results along with other published study results point to a relationship between prolonged sedentary time and increased risk for chronic conditions and premature mortality among both men and women (23,24). Reducing total sitting time and incorporating activity breaks into one’s daily schedule lowers cardiometabolic risk (25). The *American Cancer Society Guidelines on Nutrition and Physical Activity for Cancer Prevention* underscores the need to reduce total sitting time along with habitually engaging in MVPA (26). Therefore, developing and implementing programs specifically to reduce and break up sitting time at home and work is paramount. Primary care providers can play an important role in encouraging their patients to change their sedentary behavior. One study found that physicians were significantly more likely counsel their patients about the value of physical activity than to counsel them about the risks associated with sedentary behavior (27). Tools, such as the Rapid Assessment Disuse Index specifically tailored for use at the point of care, can be used by physicians to assess patients with high levels of sitting and low levels of physical activity and provide pertinent and effective counseling (27). In addition, the 5As model (28), which has been used successfully to promote physical activity in primary care, can be applied to sedentary behavior counseling.

Strengths of this study include a direct estimate of CRF, a comprehensive analytic approach, and a large sample size with numerous clinical covariates. Limitations of note were the self-reported measure of sitting time (which has not been validated), characteristics of the sample, and cross-sectional study design. More specifically, participants were asked to report estimates of time spent sitting during a typical day in broad categories which could result in misclassification of the exposure. In addition, participants were generally healthy, predominantly non-Hispanic white, and well-educated. The homogeneous nature of the cohort decreased the ability to generalize these results to more diverse populations. However, the socioeconomic homogeneity of this cohort reduced the likelihood of confounding by unmeasured factors such as occupation, income, and other socioeconomic indicators known to influence health. The cross-sectional study design limited reporting to the description of associations and thus results do not imply causality.

The more men sat, the more likely they were to be obese by any definition (ie, BMI, percentage of fat, waist circumference), but no other cardiometabolic risk factors were significantly associated with sitting time. For women, after adjustment for CRF and other covariates, no significant associations were observed between sitting time and cardiometabolic risk factors. Our results support physicians who work with their male patients to control risk factors by advising them to reduce sitting time to avoid obesity and its associated health conditions. The reduction and interruption of sitting time can be an initial step in developing a total physical activity plan that includes strategies to reduce sedentary time through increases in physical activity. Assessment of the entire intensity spectrum of behaviors from sleep to vigorous-intensity physical activity will provide health professionals with the information needed to tailor physical activity plans for risk reduction and health promotion.

## Appendix Association Between Sitting Time and the Prevalence of Detrimental Levels of Other Cardiometabolic Risk Factors

**Table:**

Characteristic/Model^b^	Sitting Time^a^				
	About 100%, Odds Ratio (95% CI)	About 75%, Odds Ratio (95% CI)	About 50%, Odds Ratio (95% CI)	About 25%, Odds Ratio (95% CI)	About 0%
**Men**					
**Elevated total cholesterol (>200 mg/dL)**					
Model A	0.78 (0.48–1.27)	0.81 (0.50–1.30)	0.83 (0.52–1.34)	0.85 (0.51–1.40)	1 [Reference]
Model B	0.69 (0.41–1.17)	0.74 (0.45–1.23)	0.78 (0.47–1.30)	0.86 (0.50–1.47)	1 [Reference]
Model C	0.70 (0.41–1.18)	0.75 (0.45–1.24)	0.78 (0.47–1.31)	0.86 (0.50–1.48)	1 [Reference]
**Elevated LDL cholesterol (>100 mg/dL)**					
Model A	0.93 (0.58–1.49)	0.96 (0.60–1.52)	0.96 (0.60–1.52)	0.92 (0.56–1.50)	1 [Reference]
Model B	0.86 (0.51–1.46)	0.92 (0.55–1.54)	0.96 (0.57–1.60)	0.99 (0.58–1.60)	1 [Reference]
Model C	0.86 (0.51–1.46)	0.93 (0.56–1.55)	0.96 (0.57–1.60)	0.99 (0.58–1.71)	1 [Reference]
**Low HDL cholesterol (<40 mg/dL)**					
Model A	1.82 (0.85–3.86)	1.73 (0.82–3.64)	1.68 (0.80–3.53)	1.74 (0.80–3.77)	1 [Reference]
Model B	1.53 (0.70–3.33)	1.57 (0.73–3.37)	1.57 (0.73–3.38)	1.62 (0.73–3.59)	1 [Reference]
Model C	1.57 (0.72–3.42)	1.62 (0.75–3.48)	1.60 (0.74–3.44)	1.63 (0.74–3.62)	1 [Reference]
**Elevated triglycerides (≥150 mg/dL)**					
Model A	1.10 (0.63–1.94)	0.97 (0.56–1.68)	1.01 (0.58–1.75)	0.99 (0.56–1.77)	1 [Reference]
Model B	0.94 (0.52–1.68)	0.87 (0.49–1.54)	0.92 (0.52–1.62)	0.93 (0.51–1.69)	1 [Reference]
Model C	0.96 (0.54–1.74)	0.91 (0.51–1.61)	0.93 (0.52–1.65)	0.93 (0.51–1.71)	1 [Reference]
**Elevated glucose (≥100 mg/dL)**					
Model A	1.15 (0.68–1.95)	1.26 (0.75–2.10)	1.11 (0.66–1.86)	1.12 (0.66–1.93)	1 [Reference]
Model B	1.07 (0.62–1.84)	1.21 (0.71–2.05)	1.05 (0.62–1.79)	1.11 (0.64–1.94)	1 [Reference]
Model C	1.08 (0.62–1.86)	1.23 (0.72–2.09)	1.06 (0.62–1.81)	1.12 (0.64–1.95)	1 [Reference]
**Elevated blood pressure (≥130/85 mm Hg)**					
Model A	0.90 (0.52–1.57)	0.69 (0.40–1.19)	0.83 (0.48–1.43)	0.72 (0.40–1.28)	1 [Reference]
Model B	0.81 (0.46–1.44)	0.65 (0.37–1.13)	0.78 (0.45–1.36)	0.70 (0.39–1.27)	1 [Reference]
Model C	0.82 (0.46–1.46)	0.66 (0.38–1.15)	0.79 (0.45–1.38)	0.71 (0.39–1.28)	1 [Reference]
**Metabolic syndrome**					
Model A	1.55 (0.76–3.20)	1.40 (0.69–2.85)	1.45 (0.71–2.94)	1.20 (0.57–2.52)	1 [Reference]
Model B	1.79 (0.86–3.73)	1.64 (0.80–3.38)	1.63 (0.79–3.35)	1.30 (0.61–2.76)	1 [Reference]
Model C	1.64 (0.76–3.53)	1.62 (0.77–3.43)	1.60 (0.75–3.38)	1.28 (0.58–2.80)	1 [Reference]
**Women**					
**Elevated total cholesterol (>200 mg/dL)**					
Model A	1.04 (0.70–1.54)	0.85 (0.60–1.20)	0.92 (0.66–1.27)	0.83 (0.60–1.14)	1 [Reference]
Model B	0.88 (0.58–1.33)	0.76 (0.53–1.09)	0.86 (0.61–1.21)	0.78 (0.56–1.09)	1 [Reference]
Model C	0.85 (0.56–1.28)	0.74 (0.52–1.06)	0.84 (0.60–1.19)	0.77 (0.55–1.07)	1 [Reference]
**Elevated LDL cholesterol (>100 mg/dL)**					
Model A	1.29 (0.88–1.90)	1.04 (0.75–1.46)	1.11 (0.80–1.53)	1.05 (0.77–1.44)	1 [Reference]
Model B	0.90 (0.60–1.37)	0.81 (0.57–1.16)	0.95 (0.67–1.34)	0.95 (0.68–1.33)	1 [Reference]
Model C	0.87 (0.57–1.32)	0.78 (0.55–1.12)	0.93 (0.66–1.31)	0.93 (0.67–1.31)	1 [Reference]
**Low HDL cholesterol (<40 mg/dL)**					
Model A	1.94 (1.06–3.54)	1.70 (0.97–2.94)	1.31 (0.76–2.26)	1.11 (0.64–1.93)	1 [Reference]
Model B	0.89 (0.46–1.74)	1.00 (0.55–1.80)	0.85 (0.47–1.52)	0.85 (0.47–1.52)	1 [Reference]
Model C	0.89 (0.45–1.73)	0.99 (0.55–1.79)	0.84 (0.47–1.51)	0.84 (0.47–1.51)	1 [Reference]
**Elevated triglycerides (≥150 mg/dL)**					
Model A	2.62 (1.39–4.91)	2.10 (1.17–3.76)	1.63 (0.91–2.91)	1.45 (0.81–2.60)	1 [Reference]
Model B	1.53 (0.77–3.02)	1.50 (0.81–2.79)	1.26 (0.68–2.31)	1.30 (0.71–2.38)	1 [Reference]
Model C	1.43 (0.72–2.83)	1.42 (0.76–2.64)	1.21 (0.66–2.22)	1.26 (0.69–2.32)	1 [Reference]
**Elevated glucose (≥100 mg/dL)**					
Model A	1.70 (0.92–3.16)	1.32 (0.75–2.32)	1.23 (0.72–2.10)	1.14 (0.67–1.93)	1 [Reference]
Model B	1.35 (0.70–2.60)	1.18 (0.66–2.12)	1.16 (0.68–2.00)	1.17 (0.68–2.01)	1 [Reference]
Model C	1.29 (0.67–2.49)	1.14 (0.64–2.06)	1.13 (0.65–1.96)	1.15 (0.67–1.98)	1 [Reference]
**Elevated blood pressure (≥130/85 mm Hg)**					
Model A	1.81 (0.87–3.75)	1.28 (0.65–2.52)	1.12 (0.58–2.14)	1.01 (0.53–1.93)	1 [Reference]
Model B	1.53 (0.72–3.24)	1.17 (0.59–2.33)	1.06 (0.55–2.04)	1.00 (0.52–1.92)	1 [Reference]
Model C	1.52 (0.72–3.22)	1.17 (0.58–2.32)	1.05 (0.54–2.03)	1.00 (0.52–1.92)	1 [Reference]
**Metabolic syndrome**					
Model A	5.43 (2.00–14.75)	3.20 (1.20–8.51)	2.52 (0.96–6.64)	2.08 (0.79–5.51)	1 [Reference]
Model B	4.42 (1.57–12.43)	2.68 (0.98–7.34)	2.27 (0.84–6.13)	2.17 (0.80–5.91)	1 [Reference]
Model C	2.44 (0.84–7.06)	1.66 (0.59–4.64)	1.63 (0.59–4.48)	1.80 (0.66–4.95)	1 [Reference]

Abbreviation: HDL, high-density lipoprotein; LDL, low-density lipoprotein; METs, metabolic equivalent of tasks.

^a^ Response options were 1) almost none of the time (about 0%), 2) approximately one-quarter of the time (about 25%), 3) approximately half of the time (about 50%), 4) approximately three-quarters of the time (about 75%), and 5) almost all of the time (about 100%).

^b^ Model A, adjusted for age; model B, adjusted for age and cardiorespiratory fitness (metabolic equivalent of tasks [METs]); and model C, adjusted for all covariates in model B plus physical activity (MET-minutes per week), alcohol consumption (drinks per week), current smoking status, waist girth (in models with lipids, glucose, or blood pressure as the outcome), and hormone replacement therapy (women only).

## Post-Test Information

To obtain credit, you should first read the journal article. After reading the article, you should be able to answer the following, related, multiple-choice questions. To complete the questions (with a minimum 75% passing score) and earn continuing medical education (CME) credit, please go to http://www.medscape.org/journal/pcd. Credit cannot be obtained for tests completed on paper, although you may use the worksheet below to keep a record of your answers. You must be a registered user on Medscape.org. If you are not registered on Medscape.org, please click on the "Register" link on the right hand side of the website to register. Only one answer is correct for each question. Once you successfully answer all post-test questions you will be able to view and/or print your certificate. For questions regarding the content of this activity, contact the accredited provider, CME@medscape.net. For technical assistance, contact CME@webmd.net. American Medical Association’s Physician’s Recognition Award (AMA PRA) credits are accepted in the US as evidence of participation in CME activities. For further information on this award, please refer to http://www.ama-assn.org/ama/pub/about-ama/awards/ama-physicians-recognition-award.page. The AMA has determined that physicians not licensed in the US who participate in this CME activity are eligible for ***AMA PRA Category 1 Credits™***. Through agreements that the AMA has made with agencies in some countries, AMA PRA credit may be acceptable as evidence of participation in CME activities. If you are not licensed in the US, please complete the questions online, print the AMA PRA CME credit certificate and present it to your national medical association for review.

## Post-Test Questions

### Study Title: Association Between Sitting Time and Cardiometabolic Risk Factors After Adjustment for Cardiorespiratory Fitness, Cooper Center Longitudinal Study, 2010–2013

#### CME Questions

1. You are seeing a 30-year-old man who works in a complex job in the software industry. His body mass index is 28 kg/m^2^, and he complains that he has a hard time losing weight because of a busy work schedule that keeps him seated in front of his computer for many hours per day. What does previous research and the current study find regarding sedentary behaviors among US adults?
  - Median sitting time per day among all US adults is approximately 2 hours per day
  - Objective assessments with accelerometers demonstrate that nearly 90% of US adults' time is spent in sedentary time
  - Less than 2% of adults reported no sitting time in the current study
  - More than 20% of the cohort in the current study reported at least 75% of their time spent sitting
2. The patient is very concerned about the relationship between his lifestyle and possible cardiometabolic risk factors. Which of the following risk factors was not related to sitting time in any of the study analyses?
  - Waist circumference
  - Blood pressure
  - Percent body fat
  - Body mass index
3. Which of the following outcomes was most associated with sitting time in multivariate adjustment among men in the current study?
  - Body fat
  - Serum triglyceride levels
  - Serum low-density lipoprotein cholesterol levels
  - Serum glucose levels

**Table:** Evaluation

**1. The activity supported the learning objectives.**				
**Strongly Disagree**	** **	** **	** **	**Strongly Agree**
1	2	3	4	5
**2. The material was organized clearly for learning to occur.**				
**Strongly Disagree**	** **	** **	** **	**Strongly Agree**
1	2	3	4	5
**3. The content learned from this activity will impact my practice.**				
**Strongly Disagree**	** **	** **	** **	**Strongly Agree**
1	2	3	4	5
**4. The activity was presented objectively and free of commercial bias.**				
**Strongly Disagree**	** **	** **	** **	**Strongly Agree**
1	2	3	4	5
